# Importance of Selected Nutrients and Additives in the Feed of Pregnant Sows for the Survival of Newborn Piglets

**DOI:** 10.3390/ani14030418

**Published:** 2024-01-27

**Authors:** Paloma Islas-Fabila, Patricia Roldán-Santiago, Luis Alberto de la Cruz-Cruz, Ofelia Limón-Morales, Anna Dutro-Aceves, Héctor Orozco-Gregorio, Herlinda Bonilla-Jaime

**Affiliations:** 1 Programa de Doctorado en Ciencias Biológicas y de la Salud, Universidad Autónoma Metropolitana, Unidad Iztapalapa, Mexico City 09340, Mexico; paloma_islas@hotmail.com; 2 Departamento de Reproducción, Facultad de Medicina Veterinaria y Zootecnia, Universidad Nacional Autónoma de México, Avenida Universidad, Mexico City 04510, Mexico; 3 Escuela de Medicina Veterinaria y Zootecnia, Universidad del Valle de México-Coyoacán, Calzada de Tlalpan, Mexico City 04910, Mexico; ladelacruzcc@gmail.com (L.A.d.l.C.-C.); annadutro@gmail.com (A.D.-A.); 4 Departamento de Producción Agrícola y Animal, Universidad Autónoma Metropolitana, Unidad Xochimilco, Calzada del Hueso 1100, Coapa, Villa Quietud, Coyoacán, Mexico City 04960, Mexico; gohector72@yahoo.com.mx; 5 Departamento de Biología de la Reproducción, Universidad Autónoma Metropolitana, Unidad Iztapalapa, Mexico City 09340, Mexico; ofelia.limon@yahoo.com

**Keywords:** nutrition, pregnant sows, fatty acid, protein, amino acids, fiber

## Abstract

**Simple Summary:**

According to the National Research Council (NRC), during gestation, sows have higher nutritional requirements to meet their needs and those of their fetuses. Therefore, an optimal feeding strategy is essential. Despite the importance of nutrition during gestation, the impact of supplementing the diets of gestating sows with foods rich in fatty acids, protein, amino acids, and dietary fiber on their offspring has not been thoroughly explored, so empirical evidence is scarce. The objective of this review is to evaluate the effect of gestating sows’ nutrition on the survival and postnatal growth of neonate piglets. Sixty percent of the publications reviewed discussed the effect of supplementing diets with one or two of these nutrients, indicating the importance of the topic. Better overall postnatal survival and growth was found to be associated with supplementation with these nutrients during gestation. The studies mainly evaluated the effect of amino acids and fiber, likely because the former are the primary source of protein for the fetus, while the latter exerts an effect on the immune system. Additional research is needed to support these findings.

**Abstract:**

This systematic review analyzed the effect of selected nutrients and additives in the feed of pregnant sows on the survival of newborn piglets. We analyzed 720 peer-reviewed publications in English in PubMed^®^ and Web of Science^®^, dated July 2023 to January 2024, related to the effect of dietary supplementation with fatty acids and various percentages of protein, amino acids, and/or sources of dietary fiber on the offspring of gestating sows. While several papers evaluated the effect of nutrition on gestating sows, only a few delved into the distinct feeding strategies required at each stage of gestation to meet the NRC’s nutritional requirements for maternal tissue gain and postnatal neonatal survival and growth. This body of research suggests that as gestation progresses the sow’s nutritional requirements increase, as the NRC established, to satisfy their own metabolic needs and those of their fetuses. Additional research is needed to determine an optimal feeding strategy.

## 1. Introduction

According to the NRC [1], sows have higher nutritional requirements during gestation to meet their metabolic needs and those of their fetuses [2]. The demand for nutrients increases throughout gestation because sows undergo several significant changes, including fetal growth, mammary growth, and colostrum production [3,4]. Therefore, inadequate maternal nutrition in relation to the increased requirements established by the NRC [1] to maintain the highest number of fetuses in utero can result in delayed fetal growth, reduced litter uniformity, low birthweights, and a higher number of stillborn piglets [3]. According to Kim et al. [4], during the first 70 days of gestation, fetuses present limited growth, so sows need an increase of only 0.25 g of protein/day. After 70 days, however, they require a significant increase (19-fold) of 4.63 g protein/day due to the growth of the placenta and the heart, liver, and intestines of the fetuses [4,5,6]. The feeding of gestating sows is generally classified into 3 stages: (1) early gestation (days 1–28), when they normally receive 2.0 kg/day of feed (depending on body condition); (2) mid-gestation (days 29–84), when feed intake should be increased by 0.15–0.20 kg/day to meet the energy required to maintain the sow and ensure adequate body weight gain [7]; and (3) late gestation (days 85–115), when the focus shifts to fetal and mammary growth, and feed intake is usually increased by 0.3–0.5 kg/day [3,7]. As gestation progresses, optimizing nutrition becomes a key factor that can lead to greater total litter weight at birth (15.06 vs. 14.36 kg), increased weight at weaning (5.37 vs. 5.20 kg), and higher individual birthweights (1.48 vs. 1.44 kg). Likewise, it can help sows produce more piglets per litter (+0.35) and more live piglets per litter (+0.34) [3,8]. A possible explanation of why optimal alimentation improves reproductive performance is that the dam’s nutritional status affects circulating progesterone that can modify endometrial development and secretory activity, and impact the composition of the allantoic fluids that carry nutrients to the fetuses [3]. Since alimentation during gestation plays an extremely important role in fetal growth and development, and in the survival and postnatal growth of neonates, several studies have evaluated feeding strategies during gestation to determine their consequences for fetal growth and development. The aim of this review is to analyze the effect of selected nutrients and additives in the feed for pregnant sows on the survival of newborn piglets.

## 2. Materials and Methods

**Figure 1:**
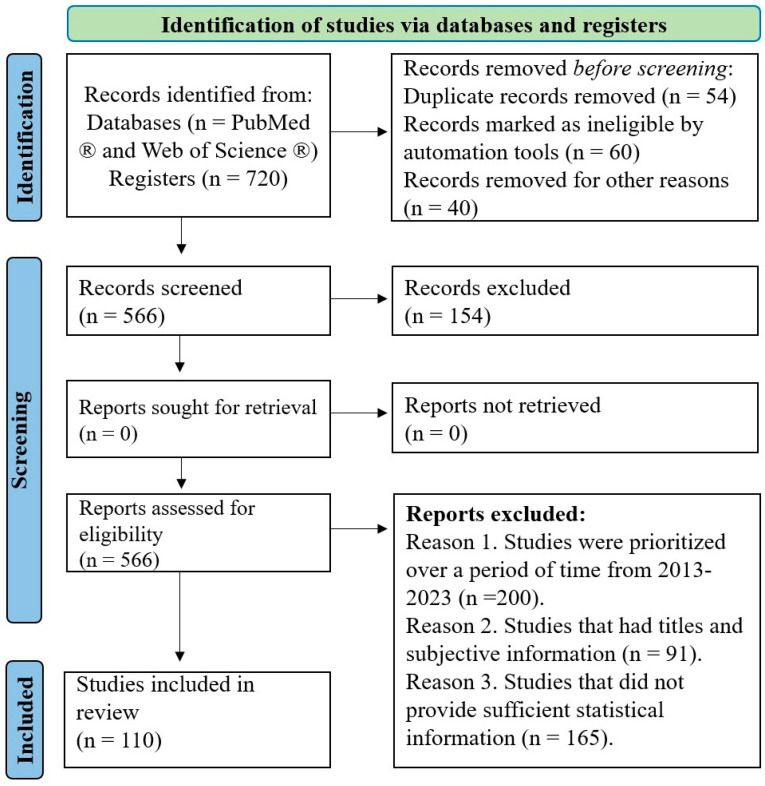
The search protocol and the resulting inclusions and exclusion. Adapted from Page et al. [9].

This systematic review was written following the Preferred Reporting Items for Systematic Reviews and Meta-Analyses (PRISMA) [9] (Figure 1).

### Exclusion Criteria

Duplicate records and records marked as ineligible by automation tools were eliminated. Studies over a time period from 2013 to 2023 were prioritized; studies that had titles and subjective information and studies that did not provide sufficient statistical information were eliminated.

#### Information Sources, Search, and Selection

We analyzed 720 peer-reviewed publications in English in PubMed^®^ and Web of Science^®^ dated July 2023 to January 2024, related to the effect of dietary supplementation with fatty acids and various percentages of protein, amino acids, and/or sources of dietary fiber on the offspring of gestating sows. The search terms were (gestating sows* OR primiparous sows* OR multiparous sows*) AND (Newborn piglets* OR neonate porcine*) AND (dietary* OR supplementation* OR additives* OR feed* OR nutritional strategies*) AND (fatty acid*) AND (protein* OR percentages of protein*) AND (amino acids* OR basic amino acids* OR several neutral amino acids*) AND (dietary fiber*). In addition, the use of * in terms allows for broadening the search results. The search terms were used in PubMed^®^ and Web of Science^®^: title, abstract and keyword (TITLE-ABS-KEY) parts of documents. EndNoteTM 20 software was used to analyze the results found in the databases.

## 3. Results and Discussion

### 3.1. Effect of Feeding throughout Gestational Periods on Offspring

During early gestation (days 1–28) the goals of providing sows with adequate nutrition are to ensure the maximum number of quality embryos and replenish the body reserves lost during previous lactations, weaning, and services [7]. In cases where sows lose considerable body reserves and exhibit poor body condition, it may be beneficial to increase the amount of feed provided during early gestation to maintain the correct metabolic and endocrine status that is vital for the development and survival of embryos and fetuses [10]. Observations show that increasing the amount of feed from 2.5 to 3.25 kg/day during early gestation in sows that present low body weight can increase litter size from 13.2 to 15.2 piglets [11].

According to Blavi et al. [12], the recommended values of standardized ileal digestible (SID) Lys and total Lys/kg of feed with a feed energy content of 12.12 MJ ME/kg are as follows: a SID Lys (g)/ME (Mcal) ratio of 1.6 is enough to satisfy the recommendations of hyperprolific sows (12–14 total piglets born), except the young animals at the end of the gestation period (85–114 days) and the multiparous highly hyperprolific (>14 total piglets born; leaner animals) ones for the period 0–85 days. The 1.9 ratio satisfies the needs of the hyperprolific gilts at the end of gestation and the highly hyperprolific throughout the first two thirds of gestation (0–85 days). The ratio should be increased to 2.3 to satisfy the requirements of highly hyperprolific sows during the last third of gestation. The recommendations for the other AA should be considered using the “Ideal Protein” concept reported in most nutrient requirement systems for swine, applied according to the recommendations of SID Lys [12].

Mid-gestation (days 29–84): during this period, sows need to increase energy inputs by 2–3 MJ/day for body maintenance and ensure adequate weight gain. This means increasing feed intake by 0.15–0.20 kg/day [10,13]. It should be noted that maternal nutrition is especially important during this stage because the formation of primary muscle fibers occurs (days 20–50 of gestation), which then serves as a template for the myogenesis of secondary muscle fibers from days 54 to 90 of gestation [10,14]. The distribution and number of muscle fibers can significantly impact the birthweight, growth, and performance of neonates, with especially large effects on daily weight gain and lean mass composition [14].

Late gestation (days 85–115): this is the period of greatest growth of the fetuses and mammary tissue, so the sow’s nutritional needs to increase substantially [10]. McPherson et al. [5] determined that fetuses require 0.25 g/d of protein up to day 69 of gestation, but that this figure increases to 4.63 g/d in late gestation. As a result, it is estimated that in the last 10 days of gestation, each fetus may gain up to one-third of its final birthweight. Consequently, meeting nutrient demands during late gestation is important to maximize fetal growth [10]. On the other hand, according to Feyera and Theil [15], from d 105 to 115 of gestation, sows require approximately 39 MJ/d of metabolizable energy and, by far, the highest proportion (79%) is lost as heat (30.5 MJ/d) [16]. The remaining 21% is retained in reproductive tissues or products, such as colostrum (3.6 MJ/d), fetal growth (2.6 MJ/d), mammary growth (1.6 MJ/d), and uterus, placenta, fluids, and membranes (0.3 MJ/d). Heat loss is required for maintenance purposes and colostrum production, fetal growth, mammary growth and growth of uterine tissues [17]. Studies show that incorporating fat into the diet in the last 10–14 days of gestation can increase the survival of swine neonates by raising birthweights from 1.36 to 1.45 kg [13]. A study by Chen et al. [18] found that feeding sows at this stage of gestation diets that do not meet the recommended energy requirements can cause piglets to exhibit lighter body weight at birth and weaning. This can reduce the weight of the small intestine and affect the height–depth relation of the crypts of the ileum and jejunum villi. Thus, it is clear that when the maternal energy requirements stipulated by the NRC [1] are not met during gestation, nutrient utilization in the growing fetus becomes selective and the development of the gastrointestinal tract may be compromised [10].

However, it is important to understand, as well, that overfeeding during late gestation can cause birth problems, such as prolonged parturition [19], likely due to a lower uterine muscle tone, especially in older sows [20]. This condition can increase the number of stillborn piglets. Moreover, even though the fetus is fully formed in late gestation, the functionality of its organ systems may be limited until a few weeks or days before birth [21], so this final maturation period is potentially an ideal time for nutrition to influence piglet quality. Other studies stress (for example, Gonçalves et al. [20] or Mallmann et al. [22]) that inadequate nutrition during gestation results in loss of body condition that may be more pronounced in this stage because, after maintenance, fetal growth is the main reason for using available nutrients. If the supply of nutrients is inadequate, the sow will mobilize body tissues to provide the nutrients needed to maintain fetal growth [23], but the manifestations of maternal tissue mobilization include reduced maternal body weight (BW) and backfat, the latter an important factor that affects the amount of colostrum, an essential element for piglet growth. Amdi et al. [24] determined that when sows have high backfat (19 mm) during gestation their piglets have higher birthweight (1.49 ± 0.02 kg; *p* < 0.05), while sows that lose backfat in late gestation tend to have low colostrum production (R^2^ = 0.12, *p* = 0.032) [25] and 25% less milk fat on day 21 of lactation [24]. Clearly, as gestation progresses, the nutritional NRC’s nutritional requirements [1] for both the dam and her fetuses change. Undoubtedly, nutrition during gestation is a main factor associated with the welfare of sows, and one that exerts a significant effect on fetal and postnatal survival, since the dam nourishes her fetuses through the placenta, and neonates through the mammary transfer system. Both delivery systems depend on appropriate nutritional intake by the dam [26]. Researchers have developed and evaluated several nutritional plans to determine the effect of supplementing the diet of gestating sows on their progeny.

### 3.2. Diets Focused on Fatty Acid Supplementation

Administering diets rich in fatty acids (fish oil and flax seed oil, among others) has been assessed, reporting that long-chain polyunsaturated fatty acids (LC-PUFA) like 20: 5n-3 (EPA), 22: 6n-3 (DHA), 22: 5n-3 (DPA), and 20: 4n-6 (ARA) participate in regulating the immune system, blood coagulation, neurotransmitters, cholesterol metabolism, and the structure of membrane phospholipids in the brain and retina [27], thus exerting important effects on fetal growth and development [28]. In contrast, a deficit of fatty acids during gestation can lead to an irreversible impairment of cognitive and/or physiological functions [28,29,30]. Similarly, administering diets rich in polyunsaturated fatty acids ensures sufficient energy intake for swine neonates [31], as these feeding regimens during late gestation and lactation increase the fat content of milk and, depending on the source of fat, modulate fatty acid profiles [32], thus favoring the development of the immune system in the early life stages of piglets. This suggests that piglets can benefit from polyunsaturated fatty acid supplementation in the sow’s diet during gestation in two ways: (1) prenatally, when developing embryos have access to docosahexaenoic acid (DHA); and (2) postpartum, when litters consume colostrum and milk with high concentrations of eicosapentaenoic acid (EPA) and DHA [33]. The work by Liu et al. [34], mentioned earlier, found that supplementing the sow’s diet with 2.5% conjugated linoleic acid (CLA) from day 85 of gestation causes a significant increase (*p* < 0.05) in colostral immunoglobulin G (IgG) concentrations, and can increase litter weight linearly (*p* < 0.05) and litter size at day 21 of lactation, while causing a linear (*p* = 0.01) decrease in pre-weaning mortality. One mechanism through which LC-PUFA may influence the growth and survival of neonates is by enhancing the immune system. Immunoglobulin G (IgG) in the colostrum is the main source of antibodies that stimulate the passive immune system of newborn piglets [27]. Another study in this field administered salmon oil at 1.79% to pregnant multiparous sows from day 105 of gestation to day 14 of lactation. The results showed that this rate of supplementation increased the total proportion of omega-3 fatty acids in the colostrum (*p* < 0.001), milk (*p* < 0.01), piglet plasma (*p* < 0.01), and adipose (*p* < 0.001), liver (*p* < 0.001), and muscle tissues (*p* < 0.001) [35]. This is important because omega-3 fatty acids play a key role in fetal brain and cognitive development, since the phospholipids that make up the cell membranes of the nervous system contain large amounts of this type of fatty acid [36].

In a separate study, 5% of hemp seeds (*Cannabis sativa*) were added to a diet from day 108 of gestation to weaning (4 weeks post-farrowing). These researchers observed that piglet body weight was influenced by this dietary treatment of the sows during the first week of lactation (2.66 vs. 3.18 kg; *p* = 0.03) [27]. Similarly, a study in which pregnant sows were supplemented with fish oil (16.5–100 g/kg) found that this type of diet reduced pre-weaning mortality rates and increased postnatal piglet growth (*p* < 0.05), mainly due to a lower number of crushed piglets and an increase in suckling behavior by the neonates [37,38]. Studies by Laws et al. [39,40] showed that supplementation with monounsaturated fatty acids (MUFA) (18:1 n-9) (100 g/kg extra) during the first semester of gestation can reduce the incidence of low-birthweight piglets (<1 kg), perhaps due to enhanced placental growth [7].

### 3.3. Diets Focused on Protein Supplementation

Proteins play roles in the body structure, nutrition, enzymatic catalysts, and molecular transport and defense of organisms, among other aspects [3]. Adding protein to the diet of gestating sows alters their metabolic characteristics [41] and impacts postnatal development and the performance of their offspring [42]. Therefore, the availability, quantity, and quality of dietary protein participate significantly in the developing embryos and fetuses [41]. For example, a 50% lower supply of dietary protein (compared to the required amount of 121 g/kg) during gestation can reduce birthweight, impair myogenesis, and restrict muscle growth potential and postnatal lean growth in neonates [43,44]. Studies also show that excessive or inadequate protein intake by the gestating sow results in a higher percentage of neonates with intrauterine growth retardation (IUGR), characterized by low birthweight (1.1 kg or less) [43]. Although newborn piglets with IUGR may experience catch-up growth after birth, they show increased adipose tissue deposition, hypercholesterolemia, reduced locomotor activity, and high mortality [45,46].

In this field, Campos et al. [3] pointed out that protein deficiency in the maternal diet (only 0.5% protein) decreases concentrations of basic amino acids (arginine, lysine, ornithine) and several neutral amino acids (alanine, glutamine, glycine, branched chain amino acids, proline, serine, taurine, threonine) in the placenta and endometrium by 16–30%, with possible negative impacts on birthweight and litter uniformity [3,47]. Similarly, a study that explored the effect of administering diets supplemented with low percentages of protein (9%) during pregnancy and lactation showed that those feeding regimens during gestation cause a significant decrease in the body weight of weaned piglets and in the daily weight gain of weaning piglets (*p* < 0.05) (Table 1) [47,48,49,50,51,52].

Studies by Jia et al. [52], meanwhile, observed that neonates from sows that ingested low protein levels (6%) exhibited low body and liver weight (*p* < 0.05). This finding is consistent with earlier reports which observed that maternal protein deprivation during gestation reduces the birthweight of piglets and decreases liver, brain, heart, and kidney weights [52,53,54]. Finally, stunted growth of piglets from gestating sows supplemented with low protein diets has been associated with low serum glucose levels and high liver glycogen at birth. Increased hepatic glycogen content suggests an adaptive mechanism of energy conservation through reduced glycolysis, or increased gluconeogenesis, in response to fetal nutritional deficiency [52]. However, it is also important to clarify that not only diets with low percentages of protein have negative effects on the sow and her progeny, but that regimens with high percentages (14–18%) can also have adverse effects on fetuses and dams, since a secondary consequence of high levels of ammonia and possibly other metabolites in plasma from a high-protein diet can create a toxic environment for both [55,56] and may reduce the size and number of skeletal muscle fibers in newborns. Regarding gestating females, an unbalanced protein intake has consequences on body weight and fat gain [44,46]. Studies emphasize that these changes can affect mammary gland development, lactation, and the interval between weaning and estrus [44]. Rehfeldt et al. [43] found that diets for gestating sows with high protein concentrations (30%) produce piglets with intrauterine growth restriction and low thymus and bone weights. As an organ of the immune system, a reduced thymus gland may be related to decreased immune function [56]. For all these reasons, the results of several studies indicate the importance of providing adequate protein levels in the diets of gestating sows.

### 3.4. Diets Focused on Amino Acid Supplementation

**Figure 2:**
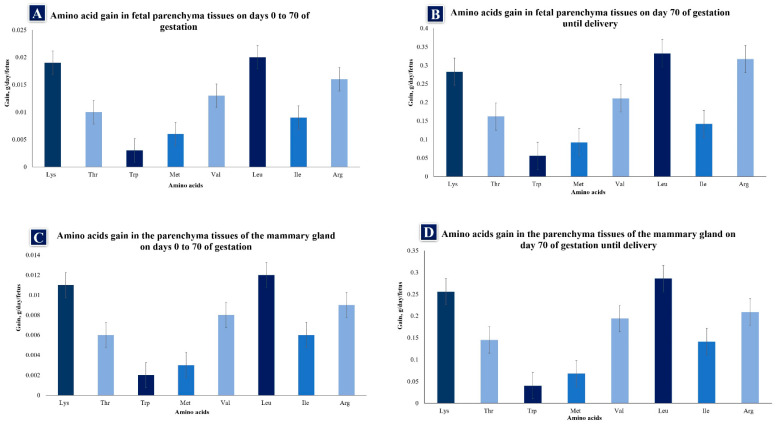
(**A**–**D**) Amino acid gain in fetal parenchymal and mammary gland tissues of gilts from day 0 of gestation to parturition. Lys = lysine, Thr = threonine, Trp = tryptophan, Met = methionine, Val = valine, Leu = leucine, Ile = isoleucine, Arg = arginine (data from Wu [59]).

The amino acid (AA) family is important in gestating sows because it regulates metabolic pathways that play fundamental roles in improving the health, survival, growth, development, lactation, and reproduction of organisms, while also participating in placental angiogenesis and placental, embryonic, and fetal development in most mammals [3,57]. According to Wu et al. [57], AAs are classified as essential or non-essential [58]. Essential AAs are defined as those of which the carbon skeletons cannot be synthesized, or are inadequately synthesized by the body relative to its needs and, hence, must be provided through the diet to meet NRC requirements [1,59,60]. Non-essential AAs are ones that the body can synthesize in adequate amounts. There is also a category of conditionally essential AAs, which the body can normally synthesize in adequate amounts, but may have to be added to the diet to meet NRC requirements [1] under conditions where utilization rates exceed synthesis rates [59]. Because mammary and fetal tissue growth is rapid during late gestation, AA needs are greater, especially in primiparous sows (Figure 2A–D). Muscle tissue growth must be taken into account among the reproductive needs of younger sows since fetal and mammary gland growth in these females occurs mainly during this stage [6], when the fetus is estimated to gain 17.5 g of protein in body tissues from day 0 to 70 (0.25 g protein/day) and 203.7 g from day 70 to 114 (4.63 g protein/day). If a sow has 14 fetuses, protein gain is 3.5 g/d and 64.8 g/d for early and late gestation, a difference of 61.3 g/d, or an 18.5-fold increase in the rate of tissue protein gain between early and late gestation [5,6]. Thus, as gestation progresses, the composition of AA varies as a consequence of changes in the rate and composition of tissue gain for fetal growth [6]. For example, observations of tryptophan (Trp) show that the supplementation of this AA during gestation reduces fetal mortality while promoting viability [61,62], perhaps because this AA serves as a precursor of several molecules (serotonin, melatonin kynurenic acid, etc. [63]) and scavenging free radicals, reactive nitrogen species, and chlorine, so it limits cellular damage [62]. During gestation, glutamine also plays a role in the immune response, and in fetal growth, survival, and metabolic regulation [64], while leucine is a key element for the development of blastocysts that can proceed to embryonic implantation [65,66].

One AA widely used in dietary supplementation of pregnant sows is arginine (Arg), an essential element for fetal growth. Arginine exists in especially high levels during early gestation in porcine allantoic fluid (4–6 mM) and can be metabolized to nitric oxide (NO) in animal cells. Nitric oxide functions as an endothelium-derived relaxing factor, neurotransmitter, and modulator of immune responses [2,44], indicating its significant metabolic role in fetal development, as a decrease in this AA during gestation can reduce NO synthesis and may alter angiogenesis and placental and endometrial tissue growth. A low Arg concentration in the placenta can reduce placental–fetal blood flow and the supply of nutrients from the dam to the fetus, ultimately delaying fetal growth [2]. Studies by Che et al. [67] demonstrated that sows fed a diet supplemented with Arg (1% L-arginine HCl up to day 114 of gestation) produced more live piglets (+1.6 piglets, *p* < 0.05) and higher total litter weight (+1.6–2.1 kg, *p* < 0.05), indicating that Arg has an important effect on fetal growth during late gestation [2,67]. Another study showed that Arg may be physiologically necessary during late gestation by playing a critical role in increasing placental angiogenesis, since extreme vascular growth and proliferation in the placenta and increased placental angiogenesis in that period allow for sufficient placental (or umbilical) blood flow and nutrient transfer for rapid fetal growth [67,68].

A recent study by Nuntapaitoon et al. [69] showed that supplementation with 0.5% L-arginine HCl reduced the proportion of piglets with restricted growth and increased the proportion of neonates with birthweights > 1.35 kg (*p* < 0.05). It is likely that these dietary effects are due to an increase in placental blood flow that allowed for more nutrients and oxygen to be transferred across the placenta. High birthweight in piglets is advantageous for survival rates during lactation [69]. In this regard, Mateo et al. [70] found that on day 7 of lactation, milk yield and the concentrations of most AAs in mother’s milk were higher in response to Arg supplementation during lactation compared to a control group (*p* < 0.05). This increase could be due to the positive effect of L-arginine on vascularization, which improves blood flow and makes nutrient absorption by the lactating mammary gland more efficient [44]. Moreover, supplementation with 0.4% of Arg from day 30 to day 114 of gestation has been shown to cause a variation of 24% in the birthweights of liveborn piglets and 22% in the proportion of live-born piglets with birthweights of 1.29 kg (*p* < 0.05) [56,59].

Lysine is considered the primary limiting AA in diets for lactating sows based on cereals and soy [71]. According to Hojgaard et al. [72], estimates of the dietary requirements of digestible standardized ileal Lys for lactating sows vary widely, from 27 to 70 g/d, or from 4.9 to 10.5 g/kg, because factors like genetics, age, litter size, appetite, and feed ingredients can all affect the dietary requirement for Lys [71,72]. Liu et al. [73] affirmed that primiparous sows eat 10–15% less than multiparous ones, so the percentage of SID Lys consumed during lactation must be increased in the former compared to the latter. Administering adequate supplies of Lys during lactation allows those sows to maximize milk production and their reproductive yield [73]. It is also important to emphasize that a low ingestion of lysine during lactation can have a negative effect on the sow’s metabolic balance, secretion of reproductive hormones, and the interval between weaning and estrus while, in contrast, a high Lys consumption can improve metabolic states in sows and increase total litter weight at birth and the weight of piglets at weaning [74]. Given these findings, diverse studies have evaluated the effect of dietary supplementation with various percentages of lysine on milk production and reproductive performance in primiparous and multiparous sows (Table 2).

For example, the work by Liu et al. [73] that evaluated the effect of dietary supplementation with 0.84, 0.94, 1.04, and 1.14% of standardized ileal digestibility (SID) Lys, balanced with Met, Thr, Trp, and Val in primiparous Yorkshire sows demonstrated that lactation increased lineally with higher levels of Lys in the diet (*p* = 0.04). These authors further showed that survival rates improve when primiparous sows are fed diets that contain 1.14% of Lys during lactation (*p* = 0.04), accompanied by higher weight (*p* = 0.04) and greater weight gain in piglets at day 21 (*p* = 0.03) [73]. Another study in this area evaluated increases in the ingestion of SID Lys (11.0, 13.5, 16.0, 18.5 g/d) in primiparous and multiparous sows (22.2 and 24.3 MJ of net energy per day, respectively), showing that the percentage of liveborn piglets increased (*p* = 0.01) with a greater ingestion of SID Lys by the multiparous sows, though not the primiparous ones, due to a treatment–group interaction (*p* = 0.04) related to the percentage of stillborn piglets. These results suggest that 11 g/day of SID Lys is an adequate level for both primiparous and multiparous gestating sows, as it provided 18.5 g/day and reduced (*p* = 0.01) the rate of fetal death by 2.3 percent [78]. All these findings highlight the importance of optimal maternal nutrition during gestation and providing the correct amount of nutrients to meet the metabolic needs of sows and their fetuses.

### 3.5. Diets Focused on Dietary Fiber Supplementation

**Figure 3:**
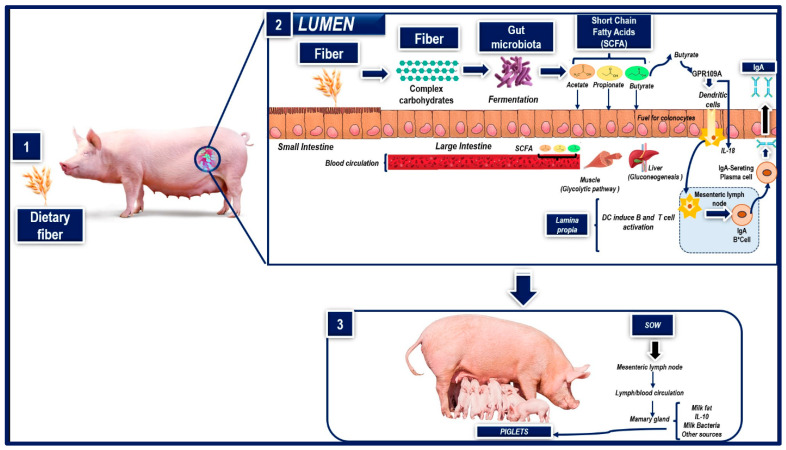
Effect of fiber on the composition of milk and colostrum. (1) Administering dietary fiber during gestation impacts colostrum quality) (2) When DF is fermented by intestinal microorganisms, it produces short-chain fatty acids (SCFAs) and (3) The sow’s mammary glands use SCFAs as precursors of milk fat synthesis which piglets consume.

Dietary fiber, generally defined as the non-digestible portion of plant-derived feeds, is a key component of many swine diets. Though not fully digested, dietary fiber can impact a wide range of physiological processes, either directly (e.g., by intestinal filling) or indirectly, by producing physiologically active gases and by-products after fermentation in the colon [79]. In addition, because dietary fibers are not hydrolyzed by endogenous enzymes in the small intestine, they are available for bacterial fermentation in the large intestine, where they can significantly modify the microbial balance with positive or negative impacts on animal health, depending on the source of the dietary fiber and the physiological state of the pig [79,80]. Adding cellulose to a standard swine diet, for example, can increase ileal populations of bifidobacteria and enterobacteria in growing pigs [80], while a selective inclusion of fiber can alter the gut microbiome and promote gut health [81]. This occurs primarily because intestinal bacteria hydrolyze dietary fibers and metabolize their constituent sugars, leading to the production of ATP [79,82]. The main end-products of microbial fermentation of dietary fiber are short-chain fatty acids (acetate, propionate, N-butyrate) and gases (carbon dioxide, hydrogen sulfide, methane) [82] (Figure 3: Part 1 and 2). Short-chain fatty acids released by anaerobic bacteria after fiber fermentation contribute to the animal’s energy supply and regulate both the growth of intestinal epithelial cells and the composition of the intestinal flora [79].

Due to the foregoing, dietary fiber (DF) supplementation in the diet of gestating sows has beneficial effects on their gut microbiota, immunity, welfare, colostrum production, physiology, and overall performance [83]. This measure can also improve farrowing and increase colostrum production [84], as the amount of feed allowable is often reduced just before farrowing, and glucose is only net-absorbed during the first 4/6 h post-feeding [84,85]. Therefore, adding dietary fiber may prove beneficial in stabilizing the post-absorption energy status in sows [86]. Another study that examined supplementation with high dietary fiber during late gestation (2 weeks before the probable date of parturition) found that this reduced the proportion of stillborn piglets from 8.8 to 6.6% (*p* < 0. 001), lowered the proportion of deaths due to low vitality (*p* < 0.001; 2.8 vs. 1.5% in the control and treatment groups, respectively), and decreased the prevalence of piglet diarrhea (*p* = 0.004; 0.7 vs. 0.3% in the control group) [84]. A study by Zhuo et al. [87] that compared multiparous sows throughout gestation (30, 60, 90 days and at birth) in relation to the supplementation of a control diet and two diets with different sources of dietary fiber, e.g., guar gum and cellulose, showed that the total number of piglets born tended to be affected by the type of diet (*p* = 0.071), as this value increased linearly in the treatments that provided sources of DF (*p* < 0.01) [84]. In addition, colostral lipid content was linearly affected by DF levels (*p* < 0.05), as the sows fed DF exhibited higher colostral lipid concentrations. Despite these benefits, however, excessive dietary fiber supplementation can decrease the birth and weaning weights of neonates. A study that evaluated four diets with different proportions of soluble fiber (diet 1: 89%; diet 2: 5.19%; diet 3: 9.12%; diet 4: 12.8%) found that litter weight at birth and average piglet weight at weaning were significantly higher in the litters of the sows that were supplemented with 3.89 and 5.19% of soluble fiber (*p* = 0.010), as both average litter weight (diet 1: 1.40 ± 0.05 kg; diet 2: 1.32 ± 0.05 kg; diet 3: 1.33 ± 0.04 kg; diet 4: 1.28 ± 0.12 kg) and piglet weight at weaning (diet 1: 7.88 ± 0.12 kg; diet 2: 7.46 ± 0.15 kg; diet 3: 6.80 ± 0.18 kg; diet 4: 6.95 ± 0.18 kg) decreased linearly as the proportion of soluble fiber increased (*p* < 0.05) [88].

It is important to note that DF supplementation impacts the composition of colostrum and milk since during gestation, a large amount of nutrients absorbed by the intestine are transported to the mammary glands through the bloodstream, so the level of nutrients in the diet affects milk and colostrum synthesis and composition in sows [89], because when DF is fermented by intestinal microorganisms, it produces short-chain fatty acids (SCFAs). The sow’s mammary glands use SCFAs as precursors of milk fat synthesis, so a high percentage of DF in their diet during gestation increases milk fat content in their colostrum (Figure 3: Part 3) [84,90]. Other observations show that administering dietary fiber during gestation affects the secretion of immunoglobulins (Ig) and interleukins (IL). In this case, a study by Shang et al. [83] compared diets with two distinct fiber sources, i.e., sugar beet pulp (SBP) and wheat bran (WB), and a control diet (corn and soybean meal), in multiparous sows at day 85 of gestation. They found that the sows fed diets supplemented with SBP had higher (*p* < 0.05) levels of immunoglobulin A (IgA) and interleukin-10 (IL-10) in their colostrum compared to the sows that received the control diet. Regarding milk composition, higher levels of IgA (*p* < 0.05) and IL-10 (*p* < 0.05) were found in the sows fed diets rich in dietary fiber (SBP and WB) compared to controls. Therefore, including dietary fiber is essential for promoting the intestinal health of piglets [83,91]. Both the third trimester of pregnancy and the lactation period are characterized by an important outflow of intestinal immune cells toward the mammary glands, since intestinal microbes can be transferred to the lymph nodes [92,93]. As a result, studies have found that certain bacteria in the intestine coexist in maternal peripheral blood and milk [94]. The dominant bacteria in sow milk are *Ruminococcaceae*, *Streptococcus*, *Lactobacillus*, and *Clostridiales*, which exist mainly in the intestine of animals [89,95]. *Ruminococcaceae* and *Lactobacillus* are especially important bacterial genera for the fermentation of dietary fiber in the intestine, so the composition of DF in the diet of pregnant sows can alter the microbial composition of her milk and increase the intestinal health of neonates [89].

Likewise, it is important to understand that dietary fiber is fermented and used by intestinal microbes to produce various metabolites, including short-chain fatty acids (SCFAs) (Figure 3: Part 1 and 2), especially acetate, propionate, and butyrate [96], which form an important substrate of gluconeogenesis and participate in regulating metabolism, immunity, and cell proliferation in sows [89,97]. Mainly short-chain fatty acids are transported to peripheral circulation through the portal vein, where they act on the liver and peripheral tissues. One proposal holds that they act as signal molecules that regulate various physiological activities of the host, such as immunity and the expression of antioxidant enzymes and inflammatory and proinflammatory factors [89]. In the mammary gland, SCFAs are transferred through the bloodstream and used as substrates for synthesizing milk fat (Figure 3: Part 3). In addition, some immune factors (e.g., IL-10) from the intestine are transported to the mammary gland through the intestinal lymphatic circulation system [89,96].

Another possibility is that intestinal microbes enter the lymph nodes through dendritic cells (DCs) in the intestinal lamina propria because DCs can phagocyte some bacterial antigens that penetrate the mucous layer, and then present them in the mesenteric lymph nodes. DCs induce B cells to differentiate into plasma cells that secrete large amounts of immunoglobulin A (IgA) in the intestinal cavity [96,97]. In addition, it has been observed that butyrate, a product of bacterial fermentation of dietary fiber, induces the expression of IL-18 in intestinal epithelial cells (IECs) through signaling via the 109 A receptor coupled to protein G (GPR109A). Likewise, butyric acid can promote the anti-inflammatory properties of colonic dendritic cells through GPR109A signaling, allowing them to induce the differentiation of Treg cells and IL-10-producing CD4+ T cells [96]. Finally, the beneficial effects of the interaction between dietary fiber and gut microbes are transmitted from sow to piglet through lactation [89].

### 3.6. Nutritional Strategies for Primiparous and Multiparous Sows

The value of post-insemination alimentary strategies in primiparous and multiparous sows has long been debated, mainly due to their potential impact on reproductive performance [22,98]. The main observations of researchers are that sows with lower parity are more sensitive to changes in body weight during lactation, and more prone to suffering later reproductive alterations. Therefore, sows use the early and mid-gestation periods to recover their body reserves [1,22]. A study that assessed the effect of increasing feed levels (1.8, 2.5, 3.2 kg/d) on early gestation in primiparous (PO1) and second-time sows (PO2) showed that those with a lower parity (PO1, PO2) and adequate body condition exhibited increases in body weight, body condition scores, and backfat (*p* < 0.001) as feed consumption increased from 1.8 to 3.2 kg/d during the first month of gestation [22,99]. However, this increase can have a negative effect on the total number of piglets born (PO1: 13.4, PO2: 15.1) likely by reducing systemic progesterone and, as a result, embryo survival [22]. Moreover, greater feed consumption (3.2 kg/d) during early gestation did not increase the number of piglets born, above all in the primiparous sows, perhaps because they required more growth to reach their target weight during their first pregnancy, since if food intake is insufficient, their bodies may prioritize growth instead of reproduction [22,100]. In this vein, a study that increased feed consumption (1.8, 2.3, 2.8, and 3.3 kg/d) in the final stage of gestation in primiparous sows found that increasing feed from day 120 of gestation to parturition increased maternal bodyweight (200.7–213.1 kg; *p* < 0.001) and the number of stillborn piglets (3.4–5.5%), but reduced feed consumption (4.2–3.9 kg; lineal: *p* = 0.001) and colostrum yield (3.6–3.2 kg) during lactation [101]. These findings concur with the work of Pedersen et al. [102], who pointed out that primiparous sows have lower colostrum yields than multiparous dams (5.2 vs. 7.1 kg; *p* < 0.01). In fact, observations at 24 h postpartum found differences in colostrum composition, as the primiparous sows had greater body fat with lower protein and casein levels than their multiparous counterparts. This suggests that the former utilize dietary nutrients differently than the latter [102,103]. Koketsu et al. [104] affirmed that primiparous sows have lower reproductive performance, likely because their endocrine system is still immature, and they have a lower capacity to consume feed. These results contrast those from Gianluppi et al.’s [105] work, which did not find greater reproductive performance or follicular size in weaned primiparous and multiparous sows that were fed 4.3 kg/day of gestation (58.78 MJ of EM and 26.66 g SID Lys) or a lactation diet (61.66 MJ of EM and 51.60 g SID Lys). These authors recommended feeding weaned sows 2.7 kg/day of a gestation diet (36.91 MJ of EM and 16.74 g SID Lys).

It Is important to emphasize, as well, that in general practice, all sows receive the same standard gestation diet and only the level of alimentation can be adjusted [106]. In most cases, the nutritional contribution of the AAs and minerals is limited, principally at the end of gestation in sows with lower parity, while excesses were observed in earlier stages and with greater frequency in higher-parity sows [107]. As a result, the development of precision feeding (PF) is providing new opportunities to identify, in real time, the factors that affect the nutritional needs of sows [106]. In this regard, models and decision support systems (DSSs) have been developed based on nutritional models that predict individual daily requirements, considering the characteristics of the animals, phases of physiological development, and housing conditions [108]. According to Gaillard et al. [106], the PF strategy makes it possible to reduce the cost of feeding by 3.6% per sow during gestation, and reduces the ingestion of nitrogen and phosphorus by 11.0 and 13.8%, respectively, and excretions by 16.7 and 15.4%, respectively, compared to sows fed under conventional alimentary systems. This suggests that the PF of gestating sows plays an important role in satisfying their requirements for amino acids while, at the same time, lowering feed costs and supplies and excretions of nitrogen and phosphorus [106]. That study, however, analyzed only one gestation cycle per sow, so it would be interesting and valuable to move beyond that to perform follow-up on the effects of PF on the performance of sows and feeding costs over various consecutive cycles, combined with the use of PF during lactation [109]. Indeed, one recent study demonstrated that applying PF during lactation also reduced feeding costs and lysine ingestion [110].

## 4. Conclusions

Given that according to the NRC, as gestation progresses, sows require greater nutritional requirements to satisfy their own metabolic needs and those of their fetuses, maternal nutrition that is inadequate for maintaining the maximal number of fetuses in the uterus can delay fetal growth, reduce litter uniformity and birthweights, and increase the number of stillborn piglets. For these reasons, numerous studies have evaluated the effect of dietary supplementation with rich amounts of fatty acids and various percentages of proteins, amino acids, and/or dietary fiber on pregnant sows and their progeny. Results show that providing dietary protein is essential for key functions such as structural roles, nutrition, enzymatic catalysts, molecular transport, and the organism’s defense system, among others. It is clear, then, that supplying protein in the diet of pregnant sows alters their metabolic characteristics. During pregnancy, amino acids regulate essential metabolic pathways for improving the health, survival, growth, development, lactation, and reproduction of organisms, while dietary fiber is crucial for the development of the microbiota and immune system of newborn piglets. Therefore, optimal feeding strategies designed for each stage of gestation must be sufficiently flexible to meet the NRC’s nutritional requirements and support both maternal tissue gain and fetal development.

## Figures and Tables

**Table 1:** Effect of diets supplemented with different percentages of protein on the reproductive performance of gestating sows.

Animals	Experimental Design	Results	Conclusion	References
59 multiparous sows (Yorkshire × Landrace) with bodyweights (BW) around 241.67 ± 8.86 kg	(1) two levels of dietary metabolizable energy (ME) density were provided (13.40 or 13.82 MJ/kg); 2) three dietary protein levels were provided from day 35 of gestation (crude protein = CP: 10.5, 12, 13.5%).	Backfat thickness in lactating sows decreased and the % of CP increased (*p* = 0.03). CP level in the diet had a negative effect on colostrum quality: % casein: *p* = 0.03; % protein: *p* = 0.04; % lactose: *p* = 0.06; total solids: *p* = 0.03; lean solids: *p* = 0.03, all decreased.	Backfat thickness and colostrum quality decreased as the CP level in the diet increased (10.5–13.5%). A diet for gestating sows containing 13.82 MJ/kg ME and 10.5% CP may improve reproductive and litter performance, and colostrum quality.	[48]
47 Landrace × Yorkshire gilts; 190 kg at insemination	Gilts were fed one of two iso-energetic compound feeds in which dietary protein differed by 12%.	Milk yield peaked at 12.9 kg/d around day 20. Sows fed the low protein compound feed had a lower milk yield from day 20 to day 40 than controls (8.0 vs. 10.3 kg/d; *p* < 0.05).	Sows on a low-protein diet had decreased milk production at the end of lactation, so it seems problematic to reduce the protein content of the lactation diet in winter, especially in gilts with limited gastric capacity.	[49]
32 Landrace × Yorkshire sows at parity two, with a similar mean bodyweight of 164.2 kg	One diet had normal crude protein (CP = 13.3%), the other had a low CP of 10.1%.	Sows receiving low levels of CP had higher serum levels of Lys and Thr and lower levels of Try, Ile, and Val (*p* < 0.05), but no effect on the serum levels of other AAs were found (*p* > 0.05).	Maternal protein deposition was decreased by a low CP.	[50]
72 F1 multiparous sows (Yorkshire × Landrace) with an average BW of 218.69 kg	Experimental diets with different CP levels, as follows: (i) CP11 containing 11% CP; (ii) CP12, 12% CP; (iii) CP13, 13% CP; (iv) CP14, 14% CP; (v) CP15, 15% CP; and (vi) CP16, 16% CP.	Increasing CP levels in the gestation diet caused a significant increase in creatinine at days 35 and 110 of gestation (linear, *p* = 0.01; linear, *p* = 0.01).	Reducing dietary CP levels from 16 to 11% in a gestation diet did not have detrimental effects on the sows’ body condition or piglet performance.	[51]

**Table 2:** Effect of diets supplemented with different percentages of lysine on the reproductive performance of primiparous and multiparous sows.

Animals	Experimental Design	Results	Conclusion	References
48 gilts (Yorkshire × Landrace), with an initial bodyweight of 168.1 ± 9.71 kg at day 35 of gestation	The first factor was metabolizable energy levels in the diet (3.265 or 3.365 kcal of ME/kg); the second was dietary lysine levels: gestation—0.55, 0.65, 0.75, and 0.85%. (total methionine 0.23%; threonine, 0.48%; tryptophan, 0.13%); Lactation—0.70, 0.85, 1, 1.15% (total methionine 0.25%; threonine 0.62%; tryptophan 0.18%).	The sows fed 3.365 kcal of EM/kg showed a tendency to present greater weight gain (*p* = 0.07). Their piglets had a higher tendency to exhibit greater weight at day 21 of lactation (*p* = 0.08). Plasma urine nitrogen levels increased as the level of lysine in the diet was raised on day 110 of gestation (*p* = 0.03).	Supplementation with lysine at 0.75% during gestation, and at 1% for lactation, with 3.365 kcal of EM/kg in primiparous sows can improve their performance and the growth of their offspring.	[74]
33 Yorkshire × Landrace multiparous sows (parities 2 and 3)	From day 90 to 110 of gestation, the sows were divided into 2 groups: control (*n* = 17) (2.6 kg/d that provided 14.8 g/d of SID Lys), and digestible ileal Lys (SID) at 40% (*n* = 16) (20.8 g/d of SID Lys, administered in soy flour).	The diets did not cause changes in the body fat or body weight of the sows in the late gestation period (*p* > 0.10), or changes in mammary tissue (*p* > 0.10).	Ingesting Lys above levels currently recommended by the NRC did not improve mammary development, so it is not necessary to use two phases to provide additional Lys protein to sows during this period.	[75]
On day 42 of gestation, 200 multiparous sows (parity = 5.1 ± 2.0) were randomly allocated to five dietary treatment groups	Experimental diets: (1) SID Lys for the mid-gestation period (days 42 to 76-indispensable amino acids). (2) SID Lys for the late gestation period (days 77 to 103-indispensable amino acids.	Total liveborn piglets per litter increased lineally and quadratically (*p* < 0.001) as the level of SID Lys in the diet increased.	Supplementation with SID Lys at 11.1 and 16.1 g/d (1.36 and 1.79 g/Mcal of metabolizable energy; 0.4% and 0.58%) for the middle and final periods of gestation, can increase the number of liveborn piglets per litter.	[76]
105 sows in their initial reproductive cycle (1.4 ± 0.5) were assigned randomly to either a precision program (PF; *n* = 50) or a control group (CON; *n* = 55)	The PF sows received two isocaloric diets (2518 kcal/kg NE; 0.80% and 0.20% standardized ileal digestible Lys [SID], respectively), while the CON sows received a diet with 0.56% SID Lys.	The sows that received the PF program had greater weight gain from day 38 to 72 (614 vs. 518 g/d; *p* < 0.05) and from day 73 to 108 (719 vs. 618 g/d; *p* = 0.063) of gestation, with greater gain in back thickness between days 63 and 110 (0.7 vs. −1.1 ± 1.6 mm; *p* < 0.05).	Using programs that include daily requirements of energy and Lys in sows during gestation helped reduce the use of feed during lactation without affecting their reproductive performance.	[77]

## Data Availability

The data presented in this study are available in this paper.
